# Machine Learning-Based Radiomics of the Optic Chiasm Predict Visual Outcome Following Pituitary Adenoma Surgery

**DOI:** 10.3390/jpm11100991

**Published:** 2021-09-30

**Authors:** Yang Zhang, Chaoyue Chen, Wei Huang, Yangfan Cheng, Yuen Teng, Lei Zhang, Jianguo Xu

**Affiliations:** 1 Department of Neurosurgery, West China Hospital, Sichuan University, Chengdu 610041, China; dryangzhang66@gmail.com (Y.Z.); chaoyuechen01@gmail.com (C.C.); yuenteng01@gmail.com (Y.T.); 2 College of Computer Science, Sichuan University, Chengdu 610065, China; weihuang@stu.edu.scu.cn; 3 Department of Neurology, West China Hospital, Sichuan University, Chengdu 610041, China; chengyfscu@163.com

**Keywords:** optic chiasm, pituitary adenoma, machine learning, radiomics, magnetic resonance imaging, visual recovery

## Abstract

Preoperative prediction of visual recovery after pituitary adenoma surgery remains a challenge. We aimed to investigate the value of MRI-based radiomics of the optic chiasm in predicting postoperative visual field outcome using machine learning technology. A total of 131 pituitary adenoma patients were retrospectively enrolled and divided into the recovery group (N = 79) and the non-recovery group (N = 52) according to visual field outcome following surgical chiasmal decompression. Radiomic features were extracted from the optic chiasm on preoperative coronal T2-weighted imaging. Least absolute shrinkage and selection operator regression were first used to select optimal features. Then, three machine learning algorithms were employed to develop radiomic models to predict visual recovery, including support vector machine (SVM), random forest and linear discriminant analysis. The prognostic performances of models were evaluated via five-fold cross-validation. The results showed that radiomic models using different machine learning algorithms all achieved area under the curve (AUC) over 0.750. The SVM-based model represented the best predictive performance for visual field recovery, with the highest AUC of 0.824. In conclusion, machine learning-based radiomics of the optic chiasm on routine MR imaging could potentially serve as a novel approach to preoperatively predict visual recovery and allow personalized counseling for individual pituitary adenoma patients.

## 1. Introduction

Pituitary adenoma is one of the most common tumors in the sellar region and commonly presents with visual disturbance due to chiasmal compression and subsequent axonal injury. Compression of the optic chiasm usually first leads to visual field (VF) defects, classically manifesting as bitemporal hemianopia [1]. Surgical resection is the predominant approach to decompress the optic chiasm and alleviate visual dysfunction, with varied degrees of visual recovery after surgery [2]. Various factors have been reported to be associated with postoperative vision recovery, including patient age, tumor size, duration of symptoms, preoperative visual function, and retinal nerve fiber layer (RNFL) thickness [3,4,5]. However, results are inconsistent across previous studies, lacking an ideal predictor that could differentiate reversible axonal injury from permanent damage. Therefore, the identification of novel markers in predicting the postoperative visual outcome is clinically valuable.

Radiomics is an emerging approach that extracts high-dimensional quantitative data from conventional medical images [6]. Furthermore, the high-throughput radiomic features could be analyzed with machine learning techniques that have shown promising potential in disease monitoring, lesion classification and prognosis prediction [7,8]. Fan et al. found that the radiomic model constructed by a support vector machine algorithm represented feasible performance in preoperative prediction of the surgical response of patients with invasive functional pituitary adenoma [9]. Zhang et al. demonstrated that MRI-based radiomics combined with random forest algorithms had the potential to predict the progression and recurrence of skull base meningiomas [10]. Recent studies have demonstrated that the microscopic radiomic parameters could reflect the underlying pathophysiological process of various diseases, especially many types of tumors [11,12]. As for optic neuropathies, previous studies have suggested that radiomic analysis of the optic nerve from magnetic resonance imaging (MRI) could potentially assess the visual outcome of patients with optic neuritis, while the utility of radiomics in compressive optic neuropathy has never been explored [13,14]. Therefore, we hypothesized that radiomic features of the optic chiasm compressed by pituitary adenomas may be associated with the underlying axonal injury and have the potential to predict vision recovery.

Thus, the purpose of the present study is to investigate whether MRI-based radiomic features of the optic chiasm could predict VF recovery after pituitary adenoma surgery using machine learning approaches. This is the first study to apply machine learning-based radiomics in prediction of visual outcome for pituitary adenoma patients. A novel radiomic model using machine learning techniques was proposed with the feasible predictive performance for postoperative visual outcome.

## 2. Materials and Methods

### 2.1. Patient Enrollment

We retrospectively reviewed patients who were pathologically confirmed with pituitary adenoma and underwent surgical chiasmal decompression at the neurosurgery department of our institution from January 2017 to June 2019. The following inclusion criteria were used: (1) chiasmal compression confirmed by preoperative sellar MRI within two weeks before operation; (2) preoperative VF defects; (3) no tumor recurrence during follow-up; (4) reliable preoperative and postoperative VF results, defined as <33% false positives responses, <33% false negative responses and <20% fixation loss. The exclusion criteria were as follows: (1) poor image quality with artifacts; (2) repeated surgery caused by tumor recurrence; (3) invisible optic chiasm in preoperative MRI images due to severe compression by pituitary adenomas; (4) history of radiotherapy or radiosurgery prior to or after surgery; (5) any other ophthalmological diseases, such as glaucoma, cataract, optic neuritis, retinal detachment or orbital tumor; (6) unexpected postoperative vision deterioration implying surgical injury (more than a 10.0 dB decrease in mean deviation) [15]. Clinical characteristics of qualified patients were collected, including age, gender, tumor diameter and surgical approach. 

This retrospective study was approved by the institutional review board of West China Hospital, Sichuan University, and the informed consent was waived (2021-S-851).

### 2.2. Ophthalmological Evaluation

The VF of all patients enrolled was evaluated with standard automated perimetry (OCTOPUS 900; Haag-Streit Inc., Köniz, Switzerland), preoperatively and postoperatively. The mean deviation (MD) was used as the VF parameter, and an MD worse than −3.0 dB was regarded as the VF defect [16,17,18]. The follow-up period after surgery ranged from 6 months to 2 years, and MD measured at the last visit was considered as the final visual outcome. Patients were divided into two groups according to the extent of VF recovery at the last follow-up: the recovery group (postoperative MD < −3.0 dB) and the non-recovery group (postoperative MD ≥ −3.0 dB) [16,17,18].

### 2.3. MRI Acquisition

All eligible participants underwent MR examinations of the sellar region with a 3.0 T scanner (Achieva, Philips, Amsterdam, The Netherlands), including T1-weighted, T2-weighted and contrast-enhanced T1-weighted images. Coronal T2-weighted imaging (T2WI) was selected for the following segmentation of the optic chiasm, given that its profile was most conspicuous in coronal T2WI. The parameters of coronal T2WI were: time repetition (TR) = 3000 ms, time echo (TE) = 80 ms, slice thickness = 2 mm, flip angle = 90°, field of view (FOV) = 230 × 230 mm^2^, voxel size = 0.45 × 0.45 × 2.2 mm^3^. 

### 2.4. Optic Chiasm Segmentation

Two researchers manually contoured the optic chiasm together slice by slice using the open-source software ITK-SNAP v3.8.0 (www.itk-snap.org, accessed on 3 April 2020) [19]. In each slice of coronal T2WI, the optic chiasm was delineated by consensus of two researchers to obtain the region of interest (ROI). The unconnected components anterior or posterior to the optic chiasm were regarded as optic nerves or optic tracts, respectively, and were not included in ROI delineation. Any discrepancy between researchers regarding the segmentation was evaluated by a senior neuroradiologist to make the final decision.

### 2.5. Radiomic Feature Extraction

Radiomic features of the segmented optic chiasm were extracted using the open-source package PyRadiomics v3.0 (https://pyradiomics.readthedocs.io/) [20]. Specifically, 14 shape features, 18 first-order features and 75 textures features (24 gray level cooccurrence matrix (GLCM) features, 16 gray level run length matrix (GLRLM) features, 16 gray level size zone matrix (GLSZM) features, 5 neighboring gray tone difference matrix (NGTDM) features and 14 gray level dependence matrix (GLDM) features) were extracted from the original images. Moreover, 744 first-order and texture features of the same type were extracted from eight wavelet transform images. Therefore, a total of 851 radiomics features were collected for each patient. The detailed definitions and formulas of these features are available at https://pyradiomics.readthedocs.io/en/latest/features.html (accessed on 3 April 2020). All radiomic features were standardized to eliminate different feature magnitudes by subtracting the mean and dividing by the standard deviation.

### 2.6. Feature Selection and Modeling

**Figure 1:**
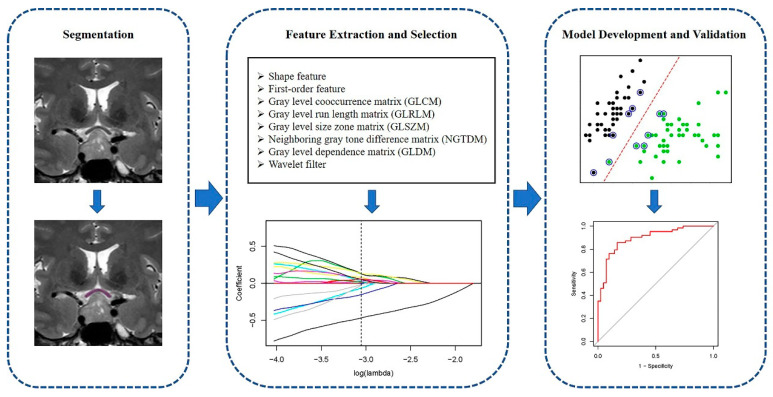
The workflow chart of the present study.

Given that high-dimensional radiomic features may contain redundant and irrelevant information, optimal features were selected first with the least absolute shrinkage and selection operator (LASSO) regression which is a commonly used feature-selection approach for high-dimensional data [21,22,23]. Then, three common machine-learning algorithms were employed to develop classification models for visual outcome, including support vector machine (SVM, https://www.rdocumentation.org/packages/e1071/versions/1.7-9/topics/svm), random forest (RF, https://www.rdocumentation.org/packages/randomForest/versions/4.6-14/topics/randomForest) and linear discriminant analysis (LDA, https://www.rdocumentation.org/packages/MASS/versions/7.3-54/topics/lda) [24]. Five-fold cross-validation was applied to evaluate the performance of models. Briefly, the dataset was randomly divided into five subsets with equal size; each subset was regarded as the validation set and the other four as the training set. Feature selection and model training were conducted on the training set; then, the predictive performance of the model was evaluated on the corresponding validation set and this process was repeated five times. Receiver operating characteristic (ROC) analysis, which is an important approach to evaluate the classification performance of the model was conducted, and the ROC curves were averaged across five folds. Area under the curve (AUC), accuracy, sensitivity, specificity, positive predict value (PPV) and negative predict value (NPV) were calculated for each model. Detailed calculation formulas of the above metrics are provided in Supplementary Material 1. All algorithms were performed with R v3.6.3. The overall workflow of this study is illustrated in Figure 1.

## 3. Results

### 3.1. Patient Characteristics

A total of 131 patients with pituitary adenoma were enrolled in this study. Among these patients, 79 (60.31%) experienced VF recovery following pituitary adenoma surgery and were classified as the recovery group, while 52 (39.69%) remained with VF defects postoperatively and were classified as the non-recovery group. The average age of patients in the recovery group and the non-recovery group was 44.81 ± 11.20 years and 49.04 ± 14.46 years, respectively (*p* = 0.078). The average MD before surgery was −8.42 ± 6.10 dB in the recovery group and −10.42 ± 6.77 dB in the non-recovery group (*p* = 0.080). The average tumor diameter in the recovery group and in the non-recovery group was 25.29 ± 5.11 mm and 26.78 ± 7.89 mm, respectively (*p* = 0.605). There were no significant differences regarding sex distribution (*p* = 0.725) and surgical approach (*p* = 0.513) between the two groups. Detailed clinical characteristics of patients are summarized in Table 1.

### 3.2. Predictive Performance of Radiomic Model

**Figure 2:**
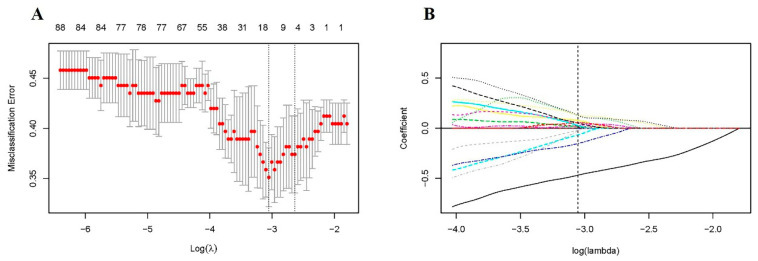
Radiomic feature selection using LASSO regression. (**A**) Tuning parameter (lambda) selection with five-fold cross-validation; (**B**) The coefficient profile plot. The vertical dotted line indicates the optimal lambda (log[lambda] = −3.052).

**Figure 3:**
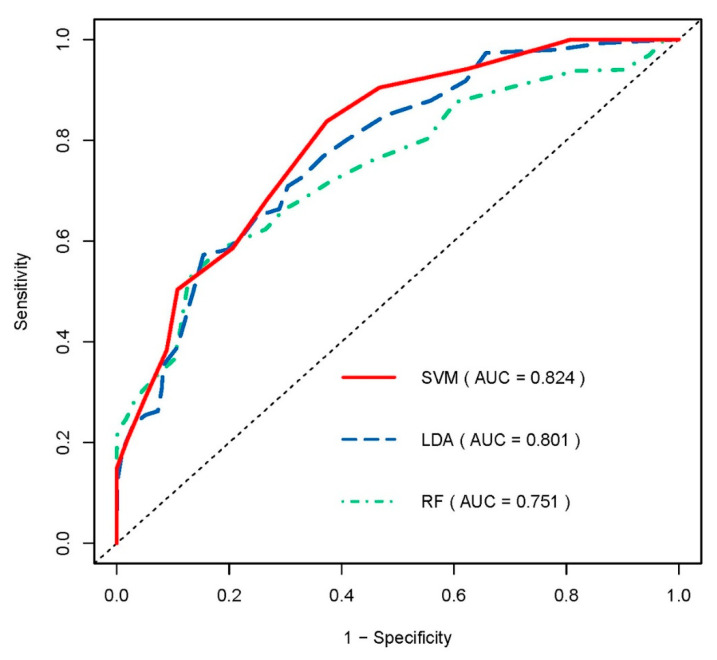
Average receiver operating characteristic (ROC) curves of three radiomic models using SVM, RF and LDA in predicting the visual field recovery based on five-fold cross-validation.

A total of 18 radiomic features were selected using LASSO regression (Figure 2). Detailed radiomic features selected for each fold are listed in Supplementary Material 2. Three machine learning models were built based on the selected radiomic features. Generally, all of the models represented feasible performances in predicting VF recovery, with an AUC of more than 0.75 in the validation set. Among the three radiomic models, the SVM-based model showed the best predictive performance, achieving the highest AUC of 0.824 in five-fold cross-validation. The AUC of the other two models based on LDA and RF was 0.801 and 0.751, respectively. Detailed performances of the three radiomic models using different machine learning algorithms are summarized in Table 2, and their average ROC curves across five-fold cross-validation are shown in Figure 3.

## 4. Discussion

Visual dysfunction is a common manifestation and also an important surgical indication of pituitary adenomas with chiasmal compression, especially VF defects in as many as 75% of patients [2]. VF recovery after decompression surgery is an important concern to clinicians, as the recovery extent varies across different patients with pituitary adenomas. Despite several factors having been investigated in previous studies, preoperative prediction of visual recovery remains a challenge [1]. An accurate predictor that could detect the microscopic changes of the damaged axons is required. In the present study, we investigated the prognostic value of MRI-based radiomic features of the optic chiasm. A novel radiomic model using machine learning algorithms was proposed with the feasible predictive performance for postoperative visual recovery, allowing personalized counseling for pituitary adenoma patients. 

Radiomics has been reported to be associated with the pathophysiological information of diseases, such as local ischemia and tissue heterogeneity [25,26,27,28]. Although most previous studies focused on various types of tumors, the potential value of radiomics in optic neuropathies has also been explored. One study on optic neuritis patients demonstrated that MRI-based radiomic features of the optic nerve were associated with visual function and visual outcome, implying that radiomic parameters may correlate with axonal integrity [13]. Another study also found that texture features of the optic nerve could assess the involvement of the optic nerve in optic neuritis patients [14]. Nevertheless, the value of radiomics in compressive optic neuropathy has never been clarified. Our results indicated that radiomics of the optic chiasm could potentially predict the visual outcome in pituitary adenoma patients, reinforcing the hypothesis that radiomic parameters could be considered as markers of pathological microstructural changes in axons such as ischemic injury and demyelination, which were important mechanisms of visual impairment due to chiasmal compression [1,29]. Moreover, the radiomic parameters were extracted from conventional T2WI that was routinely acquired in preoperative evaluation of pituitary adenoma patients, indicating that our radiomic model could potentially be utilized in clinical practice without additional cost or time.

With high-dimensional radiomic features extracted from the optic chiasm, machine learning approaches were employed to build predictive models for visual outcome. Recently, with the rapid advance in artificial intelligence, computer-aided diagnosis (CAD) has shown promising prospects in the medical field [7]. Radiomics combined with machine learning techniques has been widely applied in predicting patient prognosis and treatment response of various diseases [30,31,32]. One study suggested that the MRI-based radiomic model using an SVM algorithm could preoperatively predict the surgical response of patients with invasive functional pituitary adenoma [9]. Another study indicated that radiomic analysis in preoperative MRI combined with an RF algorithm could predict the progression and recurrence of skull base meningiomas [10]. In this study, we adopted three state-of-the-art machine learning algorithms that were commonly used in previous studies, including SVM, RF and LDA [33,34,35]. SVM constructs the optimal hyperplane in a high-dimensional space with the maximum distance between data points of each cluster [36]. RF builds a multitude of decision trees using bootstrap sampling and randomly selected variables, and predicts by taking the majority vote over all trees [37]. LDA is a linear classification approach that projects the data into lower-dimensional space to maximize the distance between two classes. Our results indicated that the SVM-based radiomic model represents better predictive performance for visual outcome compared with models using RF and LDA. Possible explanations for this result may be that SVM is not sensitive to overfitting and still effective in dealing with high-dimensional features in a relatively smaller dataset [36,38]. Moreover, SVM could detect the non-linear relationship between variables and classes using kernel functions, thus the complicated association between radiomic features and visual recovery was more likely to be modeled. More researches with larger datasets is required to validate this result.

The current study has several limitations. First, this study was a retrospective, single-center investigation, so selection bias may be present. Future prospective, multi-center studies are warranted to further confirm our results. Second, the postoperative visual outcome was not measured at the same timepoint. However, the impact on the results may be minimal since MD did not significantly change after 6 months postoperatively [15,39]. Third, our prognostic model could not be applied to patients whose optic chiasm was invisible in preoperative MRI caused by severe compression. Fourth, some prognostic factors, such as RFNL thickness, were not considered because optical coherence tomography (OCT) was not routinely conducted for pituitary adenoma patients in our institution. Future studies are required to investigate whether the incorporation of OCT parameters could improve the predictive performance of the model. Lastly, machine learning algorithms used in this study were all classical without modification. Future studies with novel algorithms are needed to further improve the accuracy of the model for better clinical application.

In conclusion, radiomics of the optic chiasm on routine MR imaging has the potential to preoperatively predict visual recovery of pituitary adenoma patients after surgical decompression. Our radiomic model developed using machine learning technology could potentially be utilized as a novel tool to assist clinicians in determining personalized counseling of individual patients with pituitary adenoma.

## Figures and Tables

**Table 1:** Clinical characteristics of patients.

Characteristics	Recovery Group	Non-Recovery Group	*p* Value
Number	79	52	
Age (year)	44.81 ± 11.20	49.04 ± 14.46	0.078
Gender, N (%)			0.725
Male	34 (43.04)	24 (46.15)	
Female	45 (56.96)	28 (53.85)	
Preoperative MD (dB)	−8.42 ± 6.10	−10.42 ± 6.77	0.080
Tumor diameter (mm)	25.29 ± 5.11	26.78 ± 7.89	0.605
Surgical approach			0.513
Transsphenoidal	75 (94.94)	47 (90.38)	
Transcranial	4 (5.06)	5 (9.62)	

Abbreviations: MD, mean deviation.

**Table 2:** Performances of machine learning-based radiomic models in predicting the visual field recovery with five-fold cross-validation in the validation set.

	AUC	Accuracy	Sensitivity	Specificity	PPV	NPV
SVM	0.824	0.747	0.675	0.798	0.696	0.795
LDA	0.801	0.718	0.598	0.797	0.674	0.750
RF	0.751	0.734	0.540	0.862	0.749	0.740

Abbreviations: SVM: support vector machine, LDA: linear discriminant analysis, RF: random forest, AUC: area under the curve, PPV: positive predict value, NPV: negative predict value.

## Data Availability

Data available on request due to privacy and ethical restrictions.

## Supplementary Materials

The following are available online at https://www.mdpi.com/article/10.3390/jpm11100991/s1, Supplementary Material 1: The calculation formula of the performance metrics; Supplementary Material 2: Radiomic features selected using LASSO regression in five-fold cross-validation.

## References

[1] Danesh-Meyer H.V., Yoon J.J., Lawlor M., Savino P.J. (2019). Visual loss and recovery in chiasmal compression. Prog. Retin. Eye Res..

[2] Muskens I.S., Zamanipoor Najafabadi A.H., Briceno V., Lamba N., Senders J.T., van Furth W.R., Verstegen M.J.T., Smith T.R.S., Mekary R.A., Eenhorst C.A.E. (2017). Visual outcomes after endoscopic endonasal pituitary adenoma resection: A systematic review and meta-analysis. Pituitary.

[3] Ho R.W., Huang H.M., Ho J.T. (2015). The influence of pituitary adenoma size on vision and visual outcomes after trans-sphenoidal adenectomy: A report of 78 cases. J. Korean Neurosurg. Soc..

[4] Park S.H., Kang M.S., Kim S.Y., Lee J.E., Shin J.H., Choi H., Kim S.J. (2021). Analysis of factors affecting visual field recovery following surgery for pituitary adenoma. Int. Ophthalmol..

[5] Lee G.I., Son K.Y., Park K.A., Kong D.S., Oh S.Y. (2020). Longitudinal changes in the retinal microstructures of eyes with chiasmal compression. Neurology.

[6] Gillies R.J., Kinahan P.E., Hricak H. (2016). Radiomics: Images Are More than Pictures, They Are Data. Radiology.

[7] Bi W.L., Hosny A., Schabath M.B., Giger M.L., Birkbak N.J., Mehrtash A., Allison T., Arnaout O., Abbosh C., Dunn I.F. (2019). Artificial intelligence in cancer imaging: Clinical challenges and applications. CA Cancer J. Clin..

[8] Hosny A., Parmar C., Quackenbush J., Schwartz L.H., Aerts H. (2018). Artificial intelligence in radiology. Nat. Rev. Cancer.

[9] Fan Y., Liu Z., Hou B., Li L., Liu X., Liu Z., Wang R., Lin Y., Feng F., Tian J. (2019). Development and validation of an MRI-based radiomic signature for the preoperative prediction of treatment response in patients with invasive functional pituitary adenoma. Eur. J. Radiol..

[10] Zhang Y., Chen J.H., Chen T.Y., Lim S.W., Wu T.C., Kuo Y.T., Ko C.-C., Su M.-Y. (2019). Radiomics approach for prediction of recurrence in skull base meningiomas. Neuroradiology.

[11] Lambin P., Leijenaar R.T.H., Deist T.M., Peerlings J., de Jong E.E.C., van Timmeren J., Sanduleanu S., LaRue R.T., Even A.J., Jochems A. (2017). Radiomics: The bridge between medical imaging and personalized medicine. Nat. Rev. Clin. Oncol..

[12] Limkin E.J., Sun R., Dercle L., Zacharaki E.I., Robert C., Reuzé S., Schernberg A., Paragios N., Deutsch E., Ferté C. (2017). Promises and challenges for the implementation of computational medical imaging (radiomics) in oncology. Ann. Oncol. Off. J. Eur. Soc. Med. Oncol..

[13] Cellina M., Pirovano M., Ciocca M., Gibelli D., Floridi C., Oliva G. (2021). Radiomic analysis of the optic nerve at the first episode of acute optic neuritis: An indicator of optic nerve pathology and a predictor of visual recovery?. La Radiologia Medica.

[14] Liu H.J., Zhou H.F., Zong L.X., Liu M.Q., Wei S.H., Chen Z.Y. (2019). MRI Histogram Texture Feature Analysis of the Optic Nerve in the Patients with Optic Neuritis. Chin. Med Sci. J..

[15] Chung Y.S., Na M., Yoo J., Kim W., Jung I.H., Moon J.H., Lee J., Kim S.H., Kim E.H. (2020). Optical Coherent Tomography Predicts Long-Term Visual Outcome of Pituitary Adenoma Surgery: New Perspectives From a 5-Year Follow-up Study. Neurosurgery.

[16] Yoo Y.J., Hwang J.M., Yang H.K., Joo J.D., Kim Y.H., Kim C.Y. (2020). Prognostic value of macular ganglion cell layer thickness for visual outcome in parasellar tumors. J. Neurol. Sci..

[17] Wang M.T.M., King J., Symons R.C.A., Stylli S.S., Meyer J., Daniell M.D., Savino P.J., Kaye A.H., Danesh-Meyer H.V. (2020). Prognostic Utility of Optical Coherence Tomography for Long-Term Visual Recovery Following Pituitary Tumor Surgery. Am. J. Ophthalmol..

[18] Lee J., Kim S.W., Kim D.W., Shin J.Y., Choi M., Oh M.C., Kim S.M., Kim E.H., Kim S.H., Byeon S.H. (2016). Predictive model for recovery of visual field after surgery of pituitary adenoma. J. Neurooncol..

[19] Yushkevich P.A., Piven J., Hazlett H.C., Smith R.G., Ho S., Gee J.C., Gerig G. (2006). User-guided 3D active contour segmentation of anatomical structures: Significantly improved efficiency and reliability. Neuroimage.

[20] van Griethuysen J.J.M., Fedorov A., Parmar C., Hosny A., Aucoin N., Narayan V., Beets-Tan R.G., Fillion-Robin J.-C., Pieper S., Aerts H.J. (2017). Computational Radiomics System to Decode the Radiographic Phenotype. Cancer Res..

[21] Joo L., Park J.E., Park S.Y., Nam S.J., Kim Y.H., Kim J.H., Kim H.S. (2021). Extensive peritumoral edema and brain-to-tumor interface MRI features enable prediction of brain invasion in meningioma: Development and validation. Neuro-Oncology.

[22] Ji G.W., Zhu F.P., Xu Q., Wang K., Wu M.Y., Tang W.W., Li X.-C., Wang X.-H. (2020). Radiomic Features at Contrast-enhanced CT Predict Recurrence in Early Stage Hepatocellular Carcinoma: A Multi-Institutional Study. Radiology.

[23] Cai J., Zheng J., Shen J., Yuan Z., Xie M., Gao M., Tan H., Liang Z.-G., Rong X., Li Y. (2020). A Radiomics Model for Predicting the Response to Bevacizumab in Brain Necrosis after Radiotherapy. Clin. Cancer Res. Off. J. Am. Assoc. Cancer Res..

[24] Ngiam K.Y., Khor I.W. (2019). Big data and machine learning algorithms for health-care delivery. Lancet Oncol..

[25] Lin A., Dey D. (2020). CT-based radiomics and machine learning for the prediction of myocardial ischemia: Toward increasing quantification. J. Nucl. Cardiol. Off. Publ. Am. Soc. Nucl. Cardiol..

[26] Hofmeister J., Bernava G., Rosi A., Vargas M.I., Carrera E., Montet X., Burgermeister S., Poletti P.-A., Platon A., Lovblad K.-O. (2020). Clot-Based Radiomics Predict a Mechanical Thrombectomy Strategy for Successful Recanalization in Acute Ischemic Stroke. Stroke.

[27] Li H., Zhu Y., Burnside E.S., Drukker K., Hoadley K.A., Fan C., Conzen S.D., Whitman G.J., Sutton E.J., Net J.M. (2016). MR Imaging Radiomics Signatures for Predicting the Risk of Breast Cancer Recurrence as Given by Research Versions of MammaPrint, Oncotype DX, and PAM50 Gene Assays. Radiology.

[28] Dercle L., Lu L., Schwartz L.H., Qian M., Tejpar S., Eggleton P., Zhao B., Piessevaux H. (2020). Radiomics Response Signature for Identification of Metastatic Colorectal Cancer Sensitive to Therapies Targeting EGFR Pathway. J. Natl. Cancer Inst..

[29] Kondo Y., Ramaker J.M., Radcliff A.B., Baldassari S., Mayer J.A., Ver Hoeve J.N., Zhang C.-L., Chiu S.-Y., Colello R.J., Duncn I.D. (2013). Spontaneous optic nerve compression in the osteopetrotic (op/op) mouse: A novel model of myelination failure. J. Neurosci. Off. J. Soc. Neurosci..

[30] Ji G.W., Zhu F.P., Xu Q., Wang K., Wu M.Y., Tang W.W., Li X.-C., Wang X.-H. (2019). Machine-learning analysis of contrast-enhanced CT radiomics predicts recurrence of hepatocellular carcinoma after resection: A multi-institutional study. EBioMedicine.

[31] Papp L., Pötsch N., Grahovac M., Schmidbauer V., Woehrer A., Preusser M., Mitterhauser M., Kiesel B., Wadsak W., Beyer T. (2018). Glioma Survival Prediction with Combined Analysis of In Vivo (11)C-MET PET Features, Ex Vivo Features, and Patient Features by Supervised Machine Learning. J. Nucl. Med..

[32] Trebeschi S., Drago S.G., Birkbak N.J., Kurilova I., Cǎlin A.M., Delli Pizzi A., Lalezari F., Lambregts D.M.J., Rohaan M.W., Parmar C. (2019). Predicting response to cancer immunotherapy using noninvasive radiomic biomarkers. Ann. Oncol. Off. J. Eur. Soc. Med. Oncol..

[33] Khorrami M., Prasanna P., Gupta A., Patil P., Velu P.D., Thawani R., Corredor G., Alilou M., Bera K., Fu P. (2020). Changes in CT Radiomic Features Associated with Lymphocyte Distribution Predict Overall Survival and Response to Immunotherapy in Non-Small Cell Lung Cancer. Cancer Immunol. Res..

[34] Oikonomou E.K., Williams M.C., Kotanidis C.P., Desai M.Y., Marwan M., Antonopoulos A.S., Thomas K.E., Thomas S., Akoumianakis I., Fan L.M. (2019). A novel machine learning-derived radiotranscriptomic signature of perivascular fat improves cardiac risk prediction using coronary CT angiography. Eur. Heart J..

[35] Elshafeey N., Kotrotsou A., Hassan A., Elshafei N., Hassan I., Ahmed S., Abrol S., Agarwal A., El Salek K., Bergamaschi S. (2019). Multicenter study demonstrates radiomic features derived from magnetic resonance perfusion images identify pseudoprogression in glioblastoma. Nat. Commun..

[36] Noble W.S. (2006). What is a support vector machine?. Nat. Biotechnol..

[37] Breiman L. (2001). Random Forests. Mach. Learn..

[38] Orrù G., Pettersson-Yeo W., Marquand A.F., Sartori G., Mechelli A. (2012). Using Support Vector Machine to identify imaging biomarkers of neurological and psychiatric disease: A critical review. Neurosci. Biobehav. Rev..

[39] Wang M.T.M., King J., Symons R.C.A., Stylli S.S., Daniell M.D., Savino P.J., Kaye A.H., Danesh-Meyer H.V. (2021). Temporal patterns of visual recovery following pituitary tumor resection: A prospective cohort study. J. Clin. Neurosci. Off. J. Neurosurg. Soc. Australas..

