# Supplementary material for: Machine Learning-Based Radiomics of the Optic Chiasm Predict Visual Outcome Following Pituitary Adenoma Surgery
Source: J Pers Med. 2021 Sep 30;11(10):991. doi: 10.3390/jpm11100991 (PMC8541242; doi:10.3390/jpm11100991)
Supplement: Supplementary file 1 [file jpm-11-00991-s001.zip › jpm-1381777-supplementary/supplementary/Supplementary Material 1.pdf]

Supplementary Material 1: The calculation formula of the performance metrics.

The accuracy is calculated with the following equation:

$$\text{Accuracy} = \frac{\text{TP} + \text{TN}}{\text{TP} + \text{TN} + \text{FP} + \text{FN}},$$

where TP is the true positives, TN the true negatives, FP the false positives, and FN the false negatives.

The sensitivity is calculated with the following equation:

$$\text{Sensitivity} = \frac{\text{TP}}{\text{TP} + \text{FN}}$$

The specificity is calculated with the following equation:

$$\text{Specificity} = \frac{\text{TN}}{\text{TN} + \text{FP}}$$

The positive predict value (PPV) is calculated with the following equation:

$$\text{PPV} = \frac{\text{TP}}{\text{TP} + \text{FP}}$$

The negative predict value (NPV)is calculated with the following equation:

$$\text{NPV} = \frac{\text{TN}}{\text{TN} + \text{FN}}$$
