# Supplementary material for: Machine Learning-Based Radiomics of the Optic Chiasm Predict Visual Outcome Following Pituitary Adenoma Surgery
Source: J Pers Med. 2021 Sep 30;11(10):991. doi: 10.3390/jpm11100991 (PMC8541242; doi:10.3390/jpm11100991)
Supplement: Supplementary file 1 [file jpm-11-00991-s001.zip › jpm-1381777-supplementary/supplementary/Supplementary Material 2.pdf]

Supplementary Material 2: Radiomic features selected using LASSO regression in five-fold cross-validation.

**Fold 1**	**Fold 2**	**Fold 3**	**Fold 4**	**Fold 5**
shape_SurfaceVolumeRatio	shape_SurfaceVolumeRatio	shape_SurfaceVolumeRatio	shape_SurfaceVolumeRatio	shape_SurfaceVolumeRatio
LLH_glcm_Idmn	LLH_glcm_Idmn	LLH_glcm_Idmn	LLH_glcm_Idmn	LLH_glcm_Idmn
LLH_gldm_LargeDependenceEmphasis	LLH_gldm_LargeDependenceEmphasis	LLH_gldm_LargeDependenceEmphasis	LLH_gldm_LargeDependenceEmphasis	LLH_gldm_LargeDependenceEmphasis
LLH_glszm_SizeZoneNonUniformity	LLH_glszm_SizeZoneNonUniformity	LLH_glszm_SizeZoneNonUniformity	LLH_glszm_SizeZoneNonUniformity	LLH_glszm_SizeZoneNonUniformity
LHL_glcm_Correlation	LHL_glcm_Correlation	LHL_glcm_Correlation	LHL_glcm_Correlation	LHL_glcm_Correlation
LHL_glszm_ZoneVariance	LHL_glszm_ZoneVariance	LHL_ngtdm_Busyness	LHH_glszm_GrayLevelNonUniformity	LHL_glszm_ZoneVariance
LHL_ngtdm_Busyness	LHL_ngtdm_Busyness	LHH_glszm_GrayLevelNonUniformity	HLL_glszm_LargeAreaHighGrayLevelEmphasis	LHH_glszm_GrayLevelNonUniformity
LHH_glszm_GrayLevelNonUniformity	LHH_glszm_GrayLevelNonUniformity	HLL_glszm_LargeAreaHighGrayLevelEmphasis	HLL_glszm_ZoneVariance	HLL_glszm_LargeAreaHighGrayLevelEmphasis
HLL_glszm_LargeAreaHighGrayLevelEmphasis	HLL_glszm_LargeAreaHighGrayLevelEmphasis	HLL_glszm_ZoneVariance	HLH_ngtdm_Busyness	HLL_glszm_ZoneVariance
HLL_glszm_ZoneVariance	HLL_glszm_ZoneVariance	HLH_ngtdm_Busyness	HHL_glcm_ClusterShade	HLH_ngtdm_Busyness
HLH_ngtdm_Busyness	HLH_ngtdm_Busyness	HHL_glcm_ClusterShade	HHL_glcm_Imc1	HHL_glcm_ClusterShade
HHL_glcm_ClusterShade	HHL_glcm_ClusterShade	HHL_glcm_Imc1	HHH_firstorder_Median	HHL_glcm_Imc1
HHL_glcm_Imc1	HHL_glcm_Imc1	HHH_firstorder_Median	HHH_glcm_InverseVariance	HHH_firstorder_Median
HHH_firstorder_Median	HHH_firstorder_Median	HHH_glcm_InverseVariance	HHH_gldm_DependenceVariance	HHH_glcm_InverseVariance
HHH_glcm_InverseVariance	HHH_glcm_InverseVariance	HHH_gldm_DependenceVariance	LLL_firstorder_Skewness	HHH_gldm_DependenceVariance
HHH_gldm_DependenceVariance	LLL_firstorder_Skewness	LLL_firstorder_Skewness	LLL_glcm_Idn	LLL_firstorder_Skewness
LLL_firstorder_Skewness	LLL_glcm_Idn	LLL_glcm_Idn		LLL_glcm_Idn
LLL_glcm_Idn				
